# Social isolation, loneliness and depression in young adulthood: a behavioural genetic analysis

**DOI:** 10.1007/s00127-016-1178-7

**Published:** 2016-02-03

**Authors:** Timothy Matthews, Andrea Danese, Jasmin Wertz, Candice L. Odgers, Antony Ambler, Terrie E. Moffitt, Louise Arseneault

**Affiliations:** MRC Social, Genetic and Developmental Psychiatry Centre, Institute of Psychiatry, Psychology and Neuroscience, King’s College London, London, UK; Department of Child and Adolescent Psychiatry, Institute of Psychiatry, Psychology and Neuroscience, King’s College London, London, UK; National and Specialist Child Traumatic Stress and Anxiety Clinic, South London and Maudsley NHS Foundation Trust, London, UK; Sanford School of Public Policy, Duke University, Durham, NC USA; Departments of Psychology and Neuroscience, Psychiatry and Behavioral Sciences, and Institute for Genome Sciences and Policy, Duke University, Durham, NC USA

**Keywords:** Social isolation, Loneliness, Depression, Behavioural genetics, Young adulthood

## Abstract

**Purpose:**

To investigate the association between social isolation and loneliness, how they relate to depression, and whether these associations are explained by genetic influences.

**Methods:**

We used data from the age-18 wave of the Environmental Risk Longitudinal Twin Study, a birth cohort of 1116 same-sex twin pairs born in England and Wales in 1994 and 1995. Participants reported on their levels of social isolation, loneliness and depressive symptoms. We conducted regression analyses to test the differential associations of isolation and loneliness with depression. Using the twin study design, we estimated the proportion of variance in each construct and their covariance that was accounted for by genetic and environmental factors.

**Results:**

Social isolation and loneliness were moderately correlated (*r* = 0.39), reflecting the separateness of these constructs, and both were associated with depression. When entered simultaneously in a regression analysis, loneliness was more robustly associated with depression. We observed similar degrees of genetic influence on social isolation (40 %) and loneliness (38 %), and a smaller genetic influence on depressive symptoms (29 %), with the remaining variance accounted for by the non-shared environment. Genetic correlations of 0.65 between isolation and loneliness and 0.63 between loneliness and depression indicated a strong role of genetic influences in the co-occurrence of these phenotypes.

**Conclusions:**

Socially isolated young adults do not necessarily experience loneliness. However, those who are lonely are often depressed, partly because the same genes influence loneliness and depression. Interventions should not only aim at increasing social connections but also focus on subjective feelings of loneliness.

## Introduction

Social relationships are a fundamental component of human life. A network of positive social relationships provides a source of support, meaning and guidance which can influence long-term trajectories of health outcomes [1]. The absence of these relationships—social isolation—is a situation that many people experience at some point in their lives, with potential implications for their health and well-being [2, 3]. Furthermore, beyond the objective absence of social relationships are differences in the way people perceive their social environments. The feeling that one’s desired quality and quantity of social connections are not being fulfilled—loneliness—constitutes an adversity in its own right. In the present study, we examined the separateness of social isolation and loneliness, and their differential associations with depressive symptoms. Further, using twin data, we investigated the underlying genetic and environmental influences that may account for some of these associations.

Social isolation is a state of estrangement, in which social connections are limited or absent. Loneliness, on the other hand, is a subjective feeling of distress, arising when social connections are perceived to be inadequate or unfulfilling [4–6]. Crucially, although isolation and loneliness tend to co-occur, they can also be experienced independently of one another: it does not follow that isolated individuals necessarily feel lonely, nor does an abundance of social connections preclude one from experiencing loneliness [7, 8]. Thus, although there is overlap between these two constructs, there are important conceptual distinctions between them. It is therefore important to incorporate measures of both isolation and loneliness, without treating them as interchangeable [5].

Loneliness is a strong risk factor for depression, over and above measures of objective social connection [9–15]. Although the prevalence of loneliness varies with age, its association with depression remains stable across the lifespan [16, 17]. However, the nature of loneliness may vary at different stages of life as individuals’ social needs shift in focus [18]. During the transition from adolescence to early adulthood, high value is attached both to close friendships and to romantic relationships. Loneliness is particularly prevalent at this stage of life [17–19], making young adulthood an interesting period in its own right for the study of loneliness and its association with social isolation and depression. We anticipate that feelings of loneliness will co-occur with greater social isolation, but that the separateness of these constructs will be reflected in only a modest association between the two. Further, based on the conceptualisation of loneliness as an emotional state, in contrast to the more circumstantial nature of isolation, we expect that loneliness will have the more robust association with depressive symptoms.

The associations between isolation and loneliness, and between loneliness and depression, may reflect common underlying genetic or environmental influences which contribute to the co-occurrence of these phenomena. Genetically-informative studies have estimated that approximately 40–50 % of the variance in loneliness is accounted for by genetic factors [20–23]. The genetic contribution to loneliness has been represented in an evolutionary framework, in which loneliness is an adaptive response to social disconnection that provides the impetus to re-integrate with social groups [9]. This suggests that social isolation is a situation that arises from the environment, and that it is the individual’s response that is genetically influenced. However, social isolation itself shows a similar degree of genetic influence to loneliness [24], raising the possibility that some of the same heritable characteristics may be involved in both of these experiences. To date, however, no multivariate behavioural genetic studies have been carried out to estimate the extent to which the associations between isolation, loneliness and depression are explained by common genetic or environmental influences. Such evidence would be informative from a clinical practice point of view, as genetically-driven associations would suggest that interventions to reduce loneliness and associated depressive symptoms should take individuals’ social perceptions into account rather than focusing efforts purely on increasing opportunities for social participation.

The perception of being cut off from social groups makes individuals feel vulnerable, triggering a range of cognitive, behavioural and physiological responses geared towards self-protection [9]. Thus, lonely individuals are inclined to be less trusting, to be more anxious and pessimistic, to perceive others around them more negatively and to approach social interactions in a defensive, hostile manner [9, 25, 26]. Although such cognitive biases and behavioural styles may serve the adaptive purpose of distancing individuals from potential threats, the corollary of this is that lonely individuals may become further isolated by sabotaging their opportunities to develop positive social relationships. It is therefore possible that a genetic predisposition to these defensive patterns of thought and behaviour, reflected in the heritability of loneliness, may also contribute to social isolation. Based on this, we would expect to find a genetic correlation between social isolation and loneliness, reflecting the presence of common underlying genetic contributions to these constructs. Similarly, in light of the negative emotional states associated with loneliness and evidence for a genetic contribution to depression [27], we expect to observe some genetic overlap between loneliness and depression.

The aim of the present study was to investigate the associations between social isolation and loneliness, and whether they differentially relate to depression, in a nationally-representative cohort of young people on the cusp of adult life. We examined the nature of these associations via three research questions: (1) To what extent are social isolation and loneliness separate constructs? (2) Are both social isolation and loneliness similarly associated with depression? (3) To what extent are the associations between isolation, loneliness and depression explained by genetic and environmental influences?

## Methods

### Participants

Participants were members of the Environmental Risk (E-Risk) Longitudinal Twin Study, which tracks the development of a birth cohort of 2232 British children. The sample was drawn from a larger birth register of twins born in England and Wales in 1994–1995 [28]. Full details about the sample are reported elsewhere [29]. Briefly, the E-Risk sample was constructed in 1999–2000, when 1116 families (93 % of those eligible) with same-sex 5-year-old twins participated in home-visit assessments. This sample comprised 55 % monozygotic (MZ) and 45 % dizygotic (DZ) twin pairs; sex was evenly distributed within zygosity (49 % male). Families were recruited to represent the UK population of families with newborns in the 1990s, on the basis of residential location throughout England and Wales and mother’s age. Teenaged mothers with twins were over-selected to replace high-risk families who were selectively lost to the register through non-response. Older mothers having twins via assisted reproduction were under-selected to avoid an excess of well-educated older mothers.

At follow-up, the study sample represents the full range of socioeconomic conditions in the UK, as reflected in the families’ distribution on a neighbourhood-level socioeconomic index (ACORN [A Classification Of Residential Neighbourhoods], developed by CACI Inc. for commercial use in Great Britain) [30]. ACORN uses census and other survey-based geodemographic discriminators to classify enumeration districts (~150 households) into socioeconomic groups ranging from “wealthy achievers” (Category 1) with high incomes, large single-family houses, and access to many amenities, to “hard-pressed” neighbourhoods (Category 5) dominated by government-subsidized housing estates, low incomes, high unemployment, and single parents. ACORN classifications were geocoded to match the location of each E-Risk study family’s home [31]. E-Risk families’ ACORN distribution closely matches that of households nation-wide: 25.6 % of E-Risk families live in “wealthy achiever” neighbourhoods compared to 25.3 % nationwide; 5.3 vs. 11.6 % live in “urban prosperity” neighbourhoods; 29.6 vs. 26.9 % live in “comfortably off” neighbourhoods; 13.4 vs. 13.9 % live in “moderate means” neighbourhoods; and 26.1 vs. 20.7 % live in “hard-pressed” neighbourhoods. E-Risk underrepresents “urban prosperity” neighbourhoods because such households are likely to be childless.

Follow-up home visits were conducted when the children were aged 7 (98 % participation), 10 (96 % participation), 12 (96 % participation), and, most recently in 2012–2014, at 18 years (93 % participation). There were 2066 children who participated in the E-Risk assessments at age 18, and the proportions of MZ (55 %) and male same-sex (47 %) twins were almost identical to those found in the original sample at age 5. The average age of the twins at the time of the assessment was 18.4 years (SD = 0.36); all interviews were conducted after the 18th birthday. There were no differences between those who did and did not take part at age 18 in terms of socioeconomic status (SES) assessed when the cohort was initially defined (*χ*^2^ = 0.86, *p* = 0.65), age-5 IQ scores (*t* = 0.98, *p* = 0.33), or age-5 internalising or externalising behaviour problems (*t* = 0.40, *p* = 0.69 and *t* = 0.41, *p* = 0.68, respectively). Home visits at ages 5, 7, 10, and 12 years included assessments with participants as well as their mother (or primary caretaker); the home visit at age 18 included interviews only with the participants. Each twin participant was assessed by a different interviewer.

The Joint South London and Maudsley and the Institute of Psychiatry Research Ethics Committee approved each phase of the study. Parents gave informed consent and twins gave assent between 5 and 12 years and then informed consent at age 18.

### Measures

The measures used in this study were administered as part of the E-Risk study’s age-18 wave of data collection. We measured social isolation via the Multidimensional Scale of Perceived Social Support (MSPSS), which assesses individuals’ access to supportive relationships with family and friends [32]. In the context of this study, we used low social support as a proxy for social isolation, as other indicators such as marital status or living alone were not applicable to the majority of 18-year olds in our sample. The 12 items in the MSPSS consist of statements such as “There is a special person who is around when I am in need” and “I can count on my friends when things go wrong”. Participants rated these statements as “not true” (0), “somewhat true” (1) or “very true” (2). We reversed the scoring of the items so that higher scores reflected disagreement with the statements. We summed scores to produce a scale with high scores reflecting greater social isolation (Cronbach *α* = 0.88).

We measured feelings of loneliness using four items from the UCLA Loneliness Scale (Version 3): [33] “How often do you feel that you lack companionship?”, “How often do you feel left out?”, “How often do you feel isolated from others?” and “How often do you feel alone?” The full UCLA Scale consists of 20 items; however, a previous study has shown that a short form of the scale has adequate validity for inclusion in large-scale studies [34]. The items were rated “hardly ever” (0), “some of the time” (1) or “often” (2). We summed the items to produce a total loneliness score (Cronbach *α* = 0.83).

We assessed current depressive symptoms using the Diagnostic Interview Schedule [35]. The interview began with four screening questions to identify participants who had experienced at least 2 weeks of persistent low mood, anhedonia or irritability in the past year, or those who had been prescribed medication for depression. Participants who answered positively to any of the screening items were asked a further 24 questions designed to map onto the nine symptoms of a major depressive episode specified in the Diagnostic and Statistical Manual of Mental Disorders, Fourth Edition (DSM-IV) [36]. We created a scale based on the total number of symptoms present. To identify participants with clinically-significant depression we used a diagnostic cut-off based on the presence of at least five symptoms plus interference in daily functioning. 20 % of participants met these criteria for a major depressive episode at 18 years.

### Data analysis

We tested the association between social isolation and loneliness using Pearson correlation. We used linear regression to test the respective associations of isolation and loneliness with depression. First, we regressed depressive symptoms separately on social isolation and loneliness. Second, we entered social isolation and loneliness simultaneously. We repeated these steps using logistic regression with a diagnosis of a major depressive episode as the dependent variable. All regression analyses were adjusted for sex and SES. As a further step in each analysis, we tested for an interaction effect between sex and the independent variables. Regression analyses were conducted in Stata 11 [37]. Participants in this study were pairs of same-sex twins, and therefore each family contained data for two children, resulting in non-independent observations. To correct for this, we used tests based on the Huber-White or sandwich variance [38], which adjusts the estimated standard errors to account for the dependence in the data.

To test genetic and environmental contributions to the relationship between social isolation, loneliness and depression, we used the twin study methodology [39]. By comparing the similarity of monozygotic (MZ) twin pairs versus dizygotic (DZ) pairs, the influences of additive genetic (A), shared environment (C) and non-shared environment (E) can be estimated. We used structural equation modelling in OpenMx [40] to fit a trivariate Cholesky decomposition in order to estimate the contributions of these influences to the covariance between social isolation, loneliness and depression. Variables were log-transformed to adjust for the non-normal distributions. The Cholesky decomposition entails a specific ordering of variables, such that each variable can be influenced by factors underlying the variables that precede it, but not vice versa. This assumes an a priori rationale for the ordering of variables, such as observations made at different time points. As all variables were measured at the same time, this assumption was not justified; therefore, the results of the initial Cholesky decomposition were transformed into the mathematically-equivalent correlated factors solution [41].

## Results

### Differential associations between social isolation, loneliness and depression in young adults

Descriptive statistics are presented in Table 1. Males reported greater social isolation than females, while females reported higher levels of depression. No sex differences were found for loneliness. Social isolation and loneliness were significantly correlated (*r* = 0.39, *p* < 0.001). A significant sex interaction was detected (*B* = 0.07, *p* = 0.001), indicating that the association between isolation and loneliness was stronger among females (*r* = 0.45, *p* < 0.001) than males (*r* = 0.35, *p* < 0.001). Among those who scored in the top 25 % range for isolation, only half (51 %) were also in the top 25 % range for loneliness. Similarly, of those who scored in the top 25 % for loneliness, only 47 % were also among the most isolated 25 % of twins.

**Table 1:** Descriptive statistics of measures and mean differences by sex

Measure	Whole sample				Males				Females				Mean difference (male–female)	
	*N*	Range	Mean	SD	*N*	Range	Mean	SD	*N*	Range	Mean	SD	*t*	*p*
Social isolation	2061	0–24	3.29	4.35	976	0–24	3.74	4.51	1085	0–24	2.87	4.15	4.56	<0.001
Loneliness	2051	0–8	1.57	1.94	973	0–8	1.51	1.93	1078	0–8	1.62	1.95	−1.39	0.17
Depression	2063	0–9	1.81	2.97	979	0–9	1.44	2.70	1084	0–9	2.13	3.16	−5.32	<0.001

*N* number, *SD* standard deviation

Depression was significantly correlated with social isolation (*r* = 0.21, *p* < 0.001) and loneliness (*r* = 0.38, *p* < 0.001). When social isolation and loneliness were entered simultaneously into a linear regression model (Table 2), the regression coefficient for social isolation remained significant but was reduced by 69 % compared to the univariate estimate, while the coefficient for loneliness was minimally affected. No sex differences were detected in the associations tested.

**Table 2:** Social isolation, loneliness, and their associations with depression

	Depressive symptom scale (B, 95 % CI)			Major depressive episode diagnosis (OR, 95 % CI)		
	Model 1	Model 2	Model 3	Model 1	Model 2	Model 3
Social isolation	**0.16 (0.12, 0.19)**	–	**0.05 (0.02, 0.09)**	**1.11 (1.08, 1.13)**	–	**1.03 (1.00, 1.06)**
Loneliness	–	**0.61 (0.54, 0.69)**	**0.56 (0.48, 0.65)**	–	**1.51 (1.42, 1.60)**	**1.46 (1.37, 1.56)**

Significant associations shown in bold

All analyses adjusted for sex, SES and non-independence of twin observations

*B* regression coefficient (unstandardised), *OR* odds ratio, *CI* confidence interval

These findings were replicated when we repeated the analyses using a clinical diagnosis of a major depressive episode as the outcome variable. When social isolation and loneliness were entered together into a logistic regression model (Table 2), the odds ratio for isolation reduced substantially although remained marginally significant, while the odds ratio for loneliness remained robust. This indicates that the association between social isolation and depression is in large part accounted for by the shared variance with loneliness.

### Genetic and environmental contributions to the associations between social isolation, loneliness and depression

When looking at the cross-twin within-trait correlations (Table 3), we found evidence for substantial additive genetic (A) influences on social isolation, loneliness and depression, reflected by higher correlations among MZ twins relative to DZ twins. MZ correlations well below 1 signify differences between genetically-identical individuals living in the same home, attributable to non-shared environment (E) influences on these traits. Conversely, the cross-twin correlations suggested only negligible shared environment (C) influences, which are indicated by a DZ correlation higher than half the MZ correlation. A similar pattern is observed when looking at the cross-twin cross-trait correlations, indicating a contribution of additive genetic and non-shared environmental influences to the covariation between isolation, loneliness and depression.

**Table 3:** Cross-twin correlations for social isolation, loneliness and depression

	Isolation (twin 1)	Loneliness (twin 1)	Depression (twin 1)
MZ twins			
Isolation (twin 2)	**0.41**	**0.25**	**0.17**
Loneliness (twin 2)	**0.25**	**0.37**	**0.21**
Depression (twin 2)	0.08	**0.22**	**0.31**
DZ twins			
Isolation (twin 2)	**0.17**	0.09	−0.01
Loneliness (twin 2)	**0.15**	**0.21**	0.09
Depression (twin 2)	0.01	0.08	**0.11**

Significant correlations shown in bold

*MZ* monozygotic, *DZ* dizygotic

**Fig. 1:**
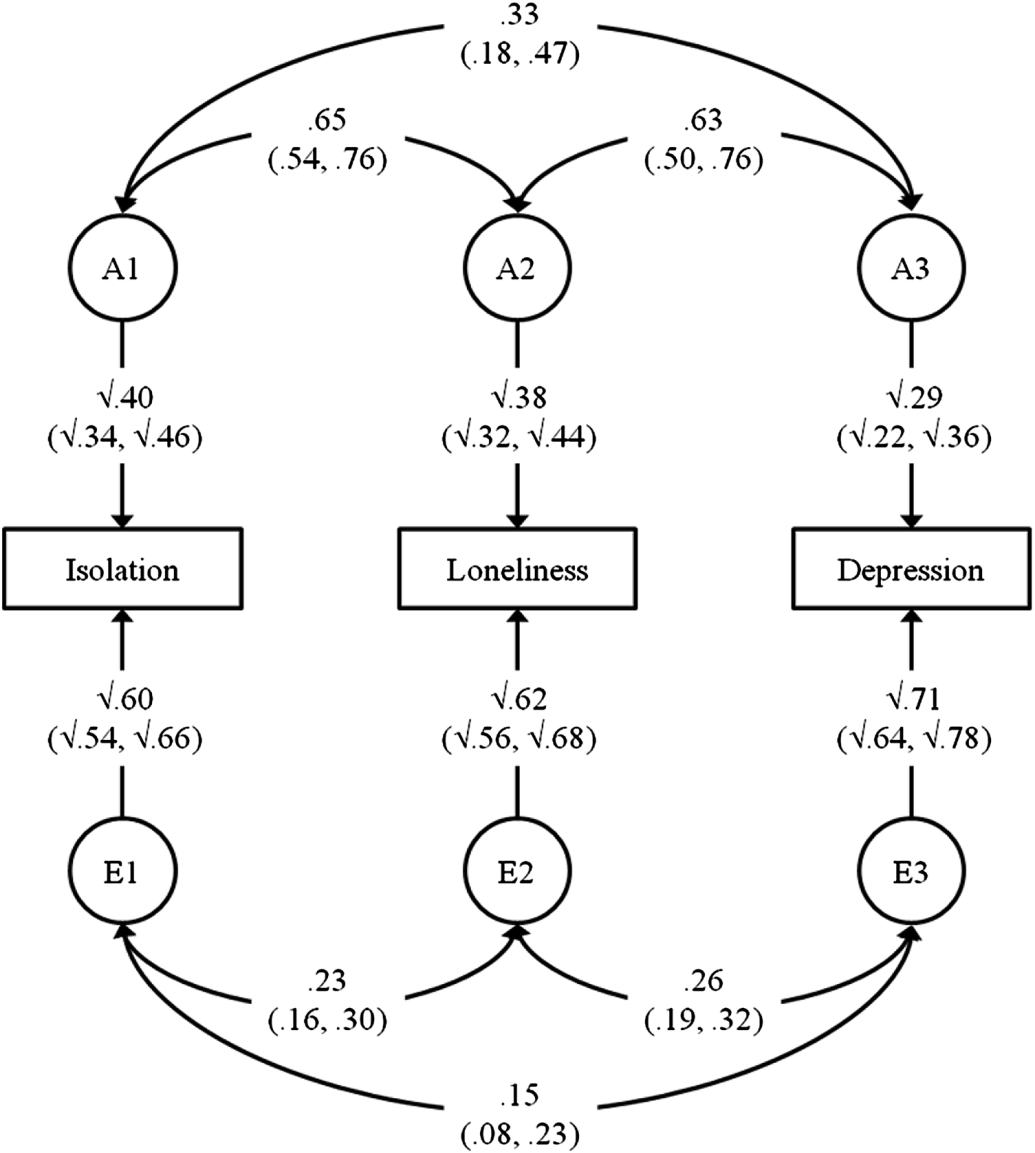
Correlated factors model separating the covariance between social isolation, loneliness and depression into additive genetic (*A*) and non-shared environment (*E*) components. *Vertical arrows* indicate the proportions of variance accounted for by the *A* and *E* factors. *Double-headed arrows* indicate the aetiological correlations between variables. 95 % confidence intervals are shown in *brackets*. The proportion of the phenotypic association between two variables that is accounted for by genetic influences can be calculated by multiplying the paths connecting the variables via their respective A factors, and dividing by the phenotypic correlation. For example, for isolation and loneliness this is calculated as (√0.40 × 0.65 × √0.38)/0.39 = 0.65

The variances of social isolation, loneliness and depression were decomposed into genetic and environmental components using behavioural genetic modelling (Fig. 1). The contribution of shared environment (C) influences could be omitted from the model without substantial loss of fit (Δ-2LL = 1.31, Δ*df* = 6, *p* = 0.97). Therefore, we present results for a more parsimonious AE model, estimating only additive genetic and non-shared environment influences. No sex differences were found for any of the estimates in the model.

Genetic influences were similar for social isolation (40 % of variance) and loneliness (38 % of variance), and slightly smaller for depression (29 % of variance). The genetic correlation between isolation and loneliness was 0.65, indicating strong overlap in the genetic influences on these constructs. The non-shared environmental correlation between isolation and loneliness was 0.23. For loneliness and depression, the genetic correlation was 0.63 and the non-shared environmental correlation was 0.26, again indicating strong genetic overlap between these variables. The genetic and non-shared environmental correlations between isolation and depression were 0.33 and 0.15, respectively.

The proportion of the phenotypic correlation between variables that is accounted for by genetic and non-shared environmental factors can be calculated using path tracing: the product of the heritability estimates for two variables and their genetic correlation yields the part of the phenotypic correlation explained by genetic influences. This can be expressed as a percentage by dividing by the phenotypic correlation. The proportion of the association between social isolation and loneliness explained by genetic influences was 65 %. When looking at loneliness and depression, genetic influences accounted for 55 % of this association, with the remainder accounted for by the non-shared environment.

## Discussion

In the present investigation, we built on previous studies in disentangling the constructs of social isolation and loneliness, using data from a nationally-representative longitudinal cohort. Young adults who were socially isolated experienced greater feelings of loneliness, and were also more likely to grapple with depression, suggesting that social relationships confer benefits for mental health over and above subjective feelings of connectedness, such as reducing the effects of stress [42]. However, young adults’ feelings of loneliness were more strongly associated with their experience of depressive symptoms than were reports of social isolation, a finding consistent with previous studies [10, 11, 15]. Using a genetically-sensitive design, we detected genetic contributions to social isolation, loneliness and depression, and a strong genetic overlap between these phenotypes.

We found a heritability estimate for loneliness which is in line with those found in previous behavioural genetics studies [20–22]. The heritability of loneliness has been described as reflecting a genetic propensity to experiencing psychological pain in conditions of social disconnection [9]. However, we also found that social isolation itself—ostensibly an environmental exposure—showed a similar degree of genetic influence to loneliness. The presence of genetic influences on measures of the environment is a robust finding in behavioural genetics research [43, 44], and in the case of social isolation may reflect heritable characteristics that predispose individuals to experience negative interactions with others, or to self-select into solitary patterns of behaviour. The absence of shared environmental influences indicates that the environmental exposures contributing to isolation and loneliness are unique to individuals rather than experienced by multiple siblings within a family.

We expanded further on previous findings on the heritability of loneliness by using a multivariate behavioural genetic design to test the hypothesis that social isolation, loneliness and depression would share common underlying genetic influences. Consistent with our expectations, the heritabilities of isolation and loneliness were highly correlated, and this genetic correlation accounted for approximately two-thirds of the phenotypic overlap between these two constructs, indicating that the co-occurrence of loneliness with social isolation is driven to a large extent by the same heritable characteristics. Some lonely individuals have a tendency to adopt negative perceptions and expectations of others, which in turn can harm their social interactions and drive others away, thus exacerbating their isolation [25, 26]. Thus, the same heritable traits that can make individuals liable to becoming isolated in the first place may also dispose them to respond to their feelings of disconnection in maladaptive ways, contributing to this self-reinforcing cycle between isolation and loneliness. A smaller part of the correlation was explained by environmental factors, which may reflect the influence of broader socioeconomic and cultural forces that shape the context in which social relationships are formed [45].

Furthermore, we found that the association between loneliness and depression was explained both by genetic and non-shared environmental influences. Although heritable personality traits such as neuroticism are correlated with both of these phenomena, other research shows that they do not explain the association between them [9, 46]. Instead, the genetic overlap may reflect a heritable predisposition to cognitive biases and negative attributional styles that are characteristics of both loneliness and depression [47]. Non-shared environmental influences, meanwhile, may be reflective of peer influences or life events. The cross-sectional nature of the data does not allow the role of mediating variables to be tested; further longitudinal research will therefore be valuable in identifying potential mechanisms underlying the associations found in this study.

The latent factor approach in this study does not yield information about which genes play a role in the associations under investigation. However, a growing body of research in this area has yielded some promising findings [23]. Studies of gene-environment interactions have found that the associations between loneliness and measures of family support were moderated by variants of genes including the serotonin transporter (5-HTTLPR) [48], the dopamine D2 receptor (DRD2) [49], and the corticotrophin-releasing hormone receptor 1 (CRHR1) [50]. Another study showed attenuation of the relationship between loneliness and depression in the presence of a specific apolipoprotein (APOE) allele [51]. Replication of these findings in large samples and research in the growing field of epigenetics will help to further elucidate the genetic underpinnings of social isolation and loneliness.

Although males were on average more isolated and females more depressed, no sex differences were found for loneliness. This is consistent with previous studies using the UCLA Loneliness Scale [52]. However, the association between isolation and loneliness was stronger among females. Previous studies suggest that friendships between females are characterised by greater amounts of emotional sharing in comparison to male friendships, which emphasise shared activities [53, 54]. To the extent that females invest more in the emotionally-supportive qualities of social relationships, this may leave them particularly susceptible to feelings of loneliness in the absence of such relationships, while males may experience this to a somewhat lesser extent. Nonetheless, it is important to note that for both males and females the association between isolation and loneliness was well below unity, indicating that non-isolated individuals may still feel lonely. Furthermore, the association between loneliness and depression was equally strong for males and females, suggesting that loneliness is a similarly distressing experience for both males and females.

In the present study, we operationalised social isolation as the lower end of a distribution of social support. Isolation has been measured in numerous others ways in different studies, including cohabitation, marital status, social network size and participation in social activities [5, 6, 11, 15, 34, 55]. There is little consensus as to the best or most comprehensive measure of isolation, and some measures may be more appropriate than others depending on the age group under investigation. For example, data on living arrangements collected at age 18 indicated that nearly all of the participants in this study were cohabiting either with family members, partners or flatmates. We therefore did not consider living alone to be a suitable measure of isolation among this age group. Other indicators of isolation were not available at age 18; however, in a previous study we derived a measure of childhood social isolation based on mother and teacher report when participants were aged 12 [24]. Repeating our analyses using this variable yielded much the same pattern of results, with 41 % of variance in social isolation accounted for by genetic influences, and approximately three-quarters of its phenotypic association with age-18 loneliness accounted for by the genetic correlation. We are therefore confident in our selection of low social support as a proxy for isolation for the purpose of this study. Nonetheless, it should be acknowledged that social support is not the only feature of social relationships that may have implications for mental health outcomes [45]. Furthermore, there may be individual differences in the way participants rate the amount of support available to them, and therefore this measure cannot be assumed to be fully objective in nature. Future studies should therefore aim to replicate our findings using measures of isolation that take into account other aspects of social networks.

Some methodological limitations in our study merit acknowledgement. Firstly, as all data were measured at the same age, our results do not permit conclusions to be drawn about the direction of the associations. Social isolation and loneliness may reinforce one another via maladaptive appraisal and coping styles, and similarly, individuals with symptoms of depression may become withdrawn and isolate themselves, feeding back into feelings of loneliness; thus, the observed associations may be bidirectional in nature. A second limitation is the use of self-report for all measures in the present study. It is not possible to rule out the presence of a reporting bias, whereby individuals with low mood are more likely to rate their social relationships more negatively. Thirdly, measuring social isolation and loneliness in a sample of twins may be confounded by the fact that each participant, by definition, had a sibling. Consequently, social isolation and loneliness may be underestimated by twin data.

With regard to clinical implications, the shared genetic origins of loneliness and depression suggest potential targets for treatment and prevention. Although the cross-sectional nature of the data does not permit any developmental hypotheses to be drawn, our findings are consistent with prior studies suggesting that interventions to decrease feelings of loneliness can be important to reduce depressive symptoms [12]. Given that loneliness can be experienced even without social isolation, simply increasing individuals’ amount of social contact may be insufficient for improving outcomes. Consistent with this, a meta-analysis of interventions suggests that addressing negative social cognitions shows greater promise as a strategy to reduce loneliness, compared to interventions focused on increasing social contact or support [56, 57]. More broadly, relationship-based interventions such as interpersonal therapy are effective in reducing depressive symptoms in young people [58].

The present study provides new insights into the links between social connection and mental health. Isolation and loneliness are strongly related constructs, and both show similar degrees of heritability. However, from a research and clinical practice perspective, it is important not to treat these constructs as interchangeable. Lonely individuals are vulnerable to depression irrespective of their actual degree of social support. Furthermore, the aetiological influences underlying these associations point to the role of common genetic characteristics in driving the co-occurrence of these experiences. To further understand the mechanisms involved, future research should investigate the role of mediating variables and gene–environment interplay in the relationship between isolation, loneliness and psychopathology.
